# Vitamin supplementation for prevention of mother-to-child transmission of HIV and pre-term delivery: a systematic review of randomized trial including more than 2800 women

**DOI:** 10.1186/1742-6405-2-4

**Published:** 2005-05-06

**Authors:** Edward J Mills, Ping Wu, Dugald Seely, Gordon H Guyatt

**Affiliations:** 1 Department of Clinical Epidemiology & Biostatistics, McMaster University, Hamilton, Canada; 2 London School of Hygiene & Tropical Medicine, London, UK; 3 Division of Clinical Epidemiology, Canadian College Of Naturopathic Medicine, Toronto, Canada; 4 Hospital for Sick Children, University of Toronto, Toronto, Canada

**Keywords:** HIV, Vitamins, Vitamin A, Mother-to-child transmission, Preterm delivery

## Abstract

**Background:**

Observational studies have suggested that low serum vitamin levels are associated with increased mother-to-child transmission (MTCT) of HIV and increased preterm delivery. We aimed to determine the efficacy of vitamins on the prevention of MTCT and preterm delivery by systematically reviewing the available randomized controlled trials [RCTs]. We conducted systematic searches of 7 electronic databases. We extracted data from the RCTs independently, in duplicate.

**Results:**

We included 4 trials in our review. Of the three trials on Vitamin A, two suggested no difference in MTCT, while the third and largest trial (*n *= 1078) suggested an increased risk of MTCT (Relative Risk 1.35, 95% Confidence Interval [CI], 1.11–1.66, P = 0.009). Two of the vitamin A trials addressed the impact of supplementation on pre-term delivery; one suggested a benefit (RR 0.65, 95% CI, 0.44–0.94) and the other no difference. All three vitamin A trials found no significant effect on infant mortality at 1 year. Of the two trials that looked at multivitamin use, only one addressed the prevention of MTCT, and found a non-significant RR of 1.04 (95% CI, 0.82–1.32). Two of the multivitamin trials found no significant effects on pre-term delivery. The single multivitamin trial examining children's mortality at 1 year yielded a non-significant RR of 0.91 (95% CI, 0.17–1.17).

**Conclusion:**

Randomized trials of vitamins to prevent MTCT have yielded conflicting results without strong evidence of benefit and have failed to exclude the possibility of harm.

## Introduction

In Africa, 55% of HIV-1-positive adults are women, most of childbearing age [1]. Data from antenatal clinics show that in several parts of southern Africa, more than 30% of pregnant women are infected with HIV-1. The fastest growth has been in South Africa, where the prevalence of infection in adults increased from 5% in 1990, to over 25% in 2002 [1]. Mother-to-child transmission (MTCT) of HIV-1 can occur during pregnancy, delivery, and post-partum through breastfeeding. In observational cohort studies, the cumulative rates of transmission are between 25% and 45% of all children born to HIV-1-infected mothers in Africa compared with 10–30% in wealthier countries [1]. This difference is greatly but not totally accounted for by the risk of postnatal transmission in populations in which breastfeeding is common.

MTCT is responsible for 5–10% of the total of new HIV infections in many developing countries, with more than 500,000 children being infected each year [1]. In many industrialized countries, the introduction of antiretroviral (ARV) drugs for the prevention of MTCT has dramatically reduced rates of transmission among non-breastfeeding mothers. Improvement is evident as more women enter pregnancy while on combination ARV therapy [2,3]. The limited access to ARV's throughout Africa has, however, led to a search for cheaper alternatives. Observational studies demonstrating an association between low biochemical and dietary levels of micronutrients and MTCT have fueled the hypothesis that micro-nutrient supplementation, particularly with Vitamin A and multivitamin combinations, may reduce vertical transmission [4-9].

Vitamin supplementation may reduce vertical transmission through either intrapartum or breastfeeding routes by reducing HIV viral load in lower genital tract secretions and in breast milk, respectively [10]. Other potential therapeutic mechanisms include improved placental and lower genital tract integrity [11], and improved fetal and newborn gastrointestinal immunity [12]. Investigators have undertaken several randomized trials addressing the impact of vitamin supplementation on MTCT. In order to determine the effectiveness of these treatments in preventing MTCT and pre-term delivery, we conducted a systematic review of these randomized trials. In addition, we addressed the effect of Vitamin A and multivitamins on childhood mortality.

## Methods

With the aid of an information specialist, we (EM, PW) performed a systematic, all language search of the following electronic databases independently, in duplicate: MedLine (1966- January 2005), AMED (1985- January 2005), AltHealthWatch (1990- January 2005), CinAhl (1982- January 2005), Embase (1980- January 2005), and the Cochrane Library (2004, issue 2). We supplemented this search by reviewing reference sections of relevant articles, and by searching for unpublished trials on the National Research Register (UK) (October 1998- January 2005) and *Clinicaltrials.gov *(February 2000- January 2005).

### Selection of abstracts

Two of us (EM, PW) independently evaluated the abstracts of retrieved articles. Eligible studies met the following criteria: (1) were original randomized controlled trials examining HIV+ patients using either Vitamin A or multivitamin treatment during pregnancy; (2) examined the outcomes of MTCT or pre-term delivery. We excluded any previous analyses of the same trial in our meta-analysis and used the most recent data available [13]. Kappa scores reflected chance-adjusted inter-observer agreement in the study identification process.

### Quality assessment

Pre-specified quality criteria included: methods of randomization, allocation concealment, blinding status of patients and assessors, use of placebo, informed consent, *a priori *sample size estimations, use of intention-to-treat, and sources of funding. In addition, we contacted the study authors for clarification of study methods.

Assessing the quality of trials included in a systematic review is important in determining trial validity, potential for introducing bias and heterogeneity and exploring subgroup analysis. Quality assessment was performed independently, in duplicate (EM, PW). Quality assessment items were used as *a priori *explanations of heterogeneity.

### Data abstraction

We extracted data independently, in duplicate (EM, PW) [14]. Data abstraction sheets were developed and piloted amongst the group (EM, PW, DS) to determine outcomes of interest and reproducibility.

### Statistical analysis

We determined agreement between reviewers using the kappa statistic. We report on study sample size and dosing using descriptive data. Outcomes measured were the number of live births, not number of pregnancies. Our primary endpoint for meta-analysis of MTCT was children's infection status at the latest time point reported. In order to provide a best-estimate of treatment effects, we conducted a meta-analysis. Our primary endpoint for the meta-analysis of pre-term delivery was pre-term delivery defined as <37 weeks. We also determined childhood mortality at 1 year where reported. All outcomes were treated as dichotomous outcomes [15] and the appropriate relative risks (RR) and applicable 95% confidence intervals [CI] were determined. We calculated RR from raw data, when provided. Pooled analysis of relative risk was conducted using a random effects model. We tested for heterogeneity using the Zalen test and the I^2 ^test [16]. *A priori *explanations of heterogeneity included quality assessment items, design, and length of follow-up. StatsDirect was used for all meta-analytic procedures (StatsDirect, Copyright 1993–2004, Manchester).

## Results

**Figure 1:**
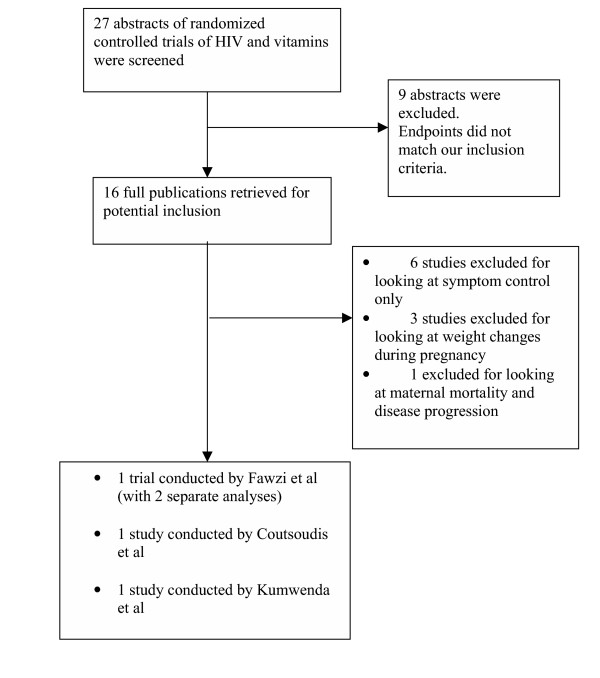
Flowchart depicting study selection and inclusion/exclusion.

Figure 1 displays the yield of our systematic searches. Of 27 clinical trial abstracts that appeared relevant, we examined 16 full text articles. Four trials met inclusion criteria and are included in this systematic review. κ for initial decisions on the inclusion of studies was 0.9, suggesting near-perfect agreement. Three [17-19] studies met our inclusion criteria of examining mother-to-child transmission of HIV and three studies [13,17,20] met our inclusion for pre-term delivery. Three studies examined the role of vitamin A for prevention of MTCT [17,18] and 1 study [19] examined the role of both vitamin A and a multivitamin using a 2 × 2 factorial design. We identified 1 unpublished and unreported study [21], from which however no results could be obtained.

### Study characteristics

Details for each of the trials can be found in table 1 (additional file 1) with regards to: the intervention; standards of care for all participants; the number of mothers randomized and the gestational period in which they were enrolled; the number of live births; compliance; outcomes measured; and results. The table is split into 2 sections to reflect details with reference to vitamin A supplementation alone or a combination of multivitamins. A brief overview of the studies found is provided here.

### Vitamin A

Fawzi et al published, in several analyses of the same factorial trial assessing the impact of vitamin A, and of multivitamins excluding Vitamin A, on vertical transmission of HIV-1 and child mortality on pregnant women in Dar es Salaam, Tanzania (n = 1078) [19]. HIV+ women presenting at antenatal clinics between 12 and 27 weeks of pregnancy were randomized to receive (i) vitamin A alone or matching placebo and, (ii) multivitamins excluding vitamin A or matching placebo. 985 children were born alive from the total sample with 898 having at least one specimen for HIV testing. Of these, 268 tested positive for HIV-1 at 6 weeks of age. Details for the earlier analyses in 1998 are also provided in Table 1 in the section on vitamin A.

In a parallel group randomized trial in Durban, South Africa, Coutsoudis et al randomized 728 pregnant HIV infected women to either placebo (n = 360) or vitamin A retinyl palmitate + B-carotene, with additional vitamin A at delivery(n = 368) [17]. Data on HIV infection at 3 months were available for 502 children of the total 661 live births.

Another parallel group trial by Kumwenda et al randomized 697 HIV infected pregnant women in Blantyre, Malawi to an intervention where vitamin A was added to their supplements (n = 340), or placebo (n = 357) [18]. There were a total of 622 live births (including 14 pairs of twins), however, 32 infants died to prior to 6 weeks of age, making HIV status undeterminable.

### Multivitamins

As described above, the factorial trial of Fawzi et al (2002) [19] performed examined the impact of both vitamin A and multivitamins of MTCT on infant mortality. The characteristics and results from the earlier analysis by Fawzi et al (1998) are also listed in table 1 [13]. In a subgroup analysis, not listed in table 1, multivitamin supplementation reduced death and prolonged HIV-free survival in women with low maternal immunological and nutritional status (RR of death 0.30, 95% CI, 0.10–0.92).

Friis et al (2004) [20] conducted a parallel randomized trial of micronutrients versus placebo. They examined a subgroup of pregnant women with HIV infection (n = 360) enrolled between the period of 22 and 36 weeks gestation (active group n = 189, control group n = 171). The study was hampered by not examining infant HIV infections or reporting specific number of births by HIV group.

### Methodological reporting

Three studies described sequence generation [13,18,20] Two reported allocation concealment [18,20]. Only 1 study described who was blinded [20]. Four studies reported obtaining informed consent [13,17,18,20]. Five studies reported an *a priori *sample size estimation [13,18-20,22] and 4 reported analysis by intention-to-treat [13,17,19,20]. All studies disclosed the sources of funding.

### Meta-analysis

**Figure 2:**
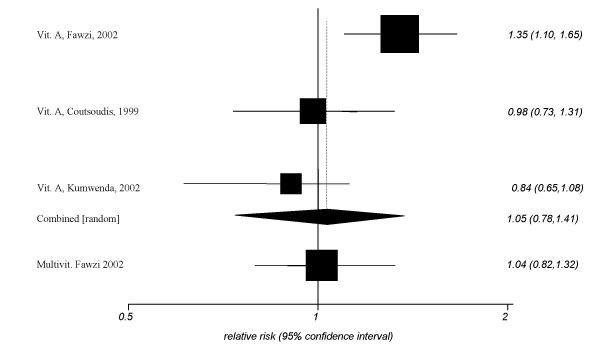
Meta-analysis of MTCT.

**Figure 3:**
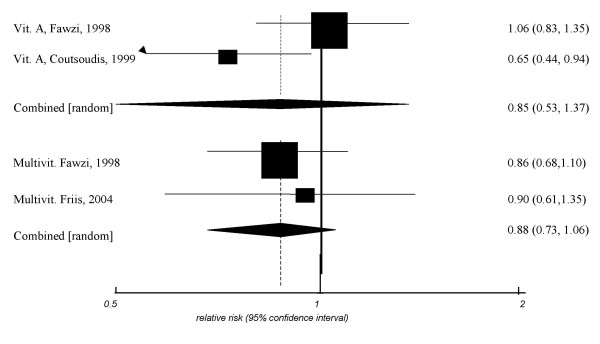
Meta-analysis of pre-term delivery.

The combined RR of vitamin A for prevention of MTCT yielded a RR of 1.05 (95% CI, 0.78–1.41, *p *= 0.2, I^2 ^= 75%, *heterogeneity P *= 0.01) (figure 2). The impressive variability in results is reflected in the largely non-overlapping confidence intervals between the two studies that suggested no difference between treatment and control, and the Fawzi study that suggested harm. Two trials examined the protection of vitamin A for pre-term delivery and yielded a non-significant pooled RR of 0.85 (95% CI, 0.53–1.37, *P *= 0.5, I^2 ^= 77%, *heterogeneity P *= 0.03) (figure 3). Three trials examined the role of maternal vitamin A supplementation on children's mortality at 1 year. The pooled RR was 1.05 (95%CI, 0.88–1.27, *P *= 0.5, I^2 ^= 0%, *heterogeneity P *= 0.8).

This single trial by Fawzi et al. examining a multivitamin for prevention of MTCT yielded a non-significant RR of 1.04 (95% CI, 0.82–1.32) (figure 2). The single trial examining maternal multivitamin intake on children's mortality at 1 year yielded a non-significant RR of 0.91 (95% CI, 0.17–1.17). Two trials examined the role of multivitamins for prevention of pre-term delivery. The combined RR yielded a non-significant RR of 0.88 (95% CI, 0.73–1.06, *P *= 0.1, I^2 ^= 0%, *heterogeneity P *= 0.8) (figure 3).

## Discussion

The results of this review should be of interest to clinicians and policy makers alike. We found that, despite early observational studies suggesting an association between vitamin A deficiency and decreased risk of MTCT [4-9], RCTs show no such effect and actually raise the possibility of increased risk. Similarly, the single trial examining supplementation with multivitamins did not decrease MTCT. Supplementation with vitamin A or multivitamins was not associated with a reduction in childhood mortality. While one trial suggested multivitamins might decrease pre-term delivery, the results are not consistent.

There are several limitations to consider in this review. Due to the small number of studies included in each separate analysis, available methods for exploring the likelihood of publication bias are uninformative. We attempted to reduce this potential impact by systematically searching the databases, contacting authors, and searching for unpublished studies through registries. A further limitation is the impact that multiple childbirths from the same mother had on the results of our analyses [23]. This information was not provided consistently across studies or through contact with authors and, although systematic evaluations of this have shown it does not significantly confound meta-analyses, could theoretically affect the estimates of effect [23]. Finally, all of the studies compared vitamin supplementation vs. placebo. It is possible that a trial examining ARVs plus micronutrients vs ARVs alone would yield results generalizable to the current desired situation.

A strength of our meta-analysis is that we used a random effects model as this assumes a different underlying effect for each study and takes between-study variability into consideration as an additional source of variation. These effects are assumed to be randomly distributed and the central point of the distribution is the focus of the combined effect estimate. Thus, the random effects model gives greater weight to smaller studies than does the fixed effects model, and results in wider confidence intervals and a more conservative estimate of effect than the fixed effects model. This is especially warranted in this study as we identified significant heterogeneity in our pooled analysis of vitamin A on MTCT (I^2 ^= 75%, *heterogeneity P *= 0.01) and pre-term delivery (I^2 ^= 77%, *heterogeneity P *= 0.03).

We found large and unexplained heterogeneity between studies in the meta-analysis of vitamin A for prevention of MTCT. We were unable to explain this heterogeneity using our *a priori *determined explanations of heterogeneity. However, biological evidence may best explain this occurrence. MTCT is most likely to occur during the process of vaginal birth. Thus, it is important that a further investigation of the trial by Fawzi et al. demonstrated that vaginal HIV-1 viral shedding actually increased in women who were given Vitamin A supplementation but not in the case of other micronutrient supplementation (74.8% vs. 65.1%, *P *= 0.04) [24]. This supports the plausibility that vitamin A may contribute to an increased risk of transmission. There does not appear to be evidence demonstrating the same risk with the use of other vitamins and multivitamins may still provide some level of protection for women living with HIV [25,26].

There is also another explanation for the difference between the trial by Fawzi et al (2002) [19] and the trial in South Africa and Malawi [17,18]. In the trials that found no effect, the supplements were given during the antenatal period only, whereas in the Tanzania trial supplementation continued during the antenatal and breastfeeding periods. It may be that a longer period of supplementation on a larger *n *resulted in greater power to detect effects. Indeed, earlier analysis of this sample by Fawzi (2000) did not reveal this effect [22]. It is additionally possible that geographical differences exist from between Tanzania and the other countries (South Africa and Malawi). It is possible that nutritional status regarding important nutritional supplementation associated with HIV progression, such as selenium [27-29], is different in Tanzania.

Conducting trials to assess the impact of interventions on MTCT is an ethically challenging, yet politically eye-opening area. Section 29 of the Helsinki Declaration ethical principles for conducting research on human subjects states that "the benefits, risks, burdens and effectiveness of a new method should be tested against those of the best current prophylactic, diagnostic, and therapeutic methods. This does not exclude the use of placebo, or no treatment, in studies where no proven prophylactic, diagnostic or therapeutic method exists [30]" However, in many impoverished nations, supplying antiretrovirals would also result in inducement to participate, a factor that is largely considered unethical to recruitment. Were antiretroviral treatment provided to these developing nation populations, vitamins would have to be tested in the presence of antiretroviral treatments, such as single-dose nevirapine or short-dose zidovudine [2]. However, access to antiretroviral treatments in developing nations is extremely limited and although the Global Fund for AIDS, Malaria and Tuberculosis is making great strides at providing access to antiretrovirals for impoverished nations, the likelihood of effective treatment even in pregnancy is not guaranteed. The investigators of the trials reviewed here have provided evidence in a pragmatic fashion as they provide results from the population with which we would aim to generalize.

More than 95% of HIV-1-infected children acquired their infection from their mother [1]. Mother-to-child transmission is largely preventable with interventions that are accessible to resource-poor countries: prevention of sexual transmission of HIV-1 through education for women of childbearing age, especially very young women; access to HIV-1 testing and reduction of unwanted pregnancies by HIV infected women informed of their serostatus; and ARV-based prevention of mother-to-child transmission. Prevention of mother-to-child transmission is the most cost-effective antiretroviral method and one of the most attractive interventions for prevention of HIV-1. A rapid scaling-up of implementation is crucial to allow programs to prevent mother-to-child transmission to affect the burden of paediatric HIV/AIDS. Such national initiatives should build a comprehensive continuum of care, including access to ARVs, for all members of affected families.

Using vitamins as a therapy to prevent MTCT seems inadvisable given the current state of evidence indicating a lack of consistent effect in prevention of vertical transmission [2,3,31]. However, in settings where poverty and social circumstances prevent adequate nutrition, the implementation of nutritional programs for pregnant women may play a role in preventing other harmful pregnancy outcomes. Future trials assessing the impact of effective nutrition on pregnant women living with HIV are not only an important effort in stemming the epidemic and improving the quality of life of patients, but also a human rights imperative [32]. Specific trials aimed at women with low nutritional status may provide an additional armament in the fight against HIV/AIDS.

In summary, the findings from our systematic review and meta-analysis do not support the use of vitamin A supplementation as an aid in reducing the risk of mother-to-child transmission of HIV-1, and may in fact increase the risk. With respect to protection against pre-term delivery, vitamin A supplementation demonstrated a non-statistically significant protective trend. No role was found for maternal vitamin A supplementation in reducing childhood mortality at 1 year. We also found that multivitamin supplementation showed no effect on mother-to-child transmission, childhood mortality at 1 year, or prevention of pre-term delivery.

## Competing interests

The author(s) declare that they have no competing interest.

## Authors' contributions

Concept, protocol: EM, PW, GG

Data searching and abstraction: EM, PW, DS

Data analysis: EM, PW, GG, DS

Manuscript drafts: EM, PW, GG, DS

Approval of final manuscript: EM, PW, GG, DS

## Supplementary Material

Additional File 1 Table 1. Study characteristics. Click here for file

## References

[B1] UNAIDS (2004). Report on the global AIDS epidemic.

[B2] Brocklehurst P (2002). Interventions for reducing the risk of mother-to-child transmission of HIV infection. Cochrane Database Syst Rev.

[B3] Brocklehurst P, Volmink J (2002). Antiretrovirals for reducing the risk of mother-to-child transmission of HIV infection. Cochrane Database Syst Rev.

[B4] Sherr L (1997). Preventing HIV transmission during pregnancy and delivery: a review. AIDS STD Health Promot Exch.

[B5] Semba RD, Miotti PG, Chiphangwi JD, Dallabetta G, Yang LP, Saah A, Hoover D (1998). Maternal vitamin A deficiency and infant mortality in Malawi. J Trop Pediatr.

[B6] Semba RD, Miotti PG, Chiphangwi JD, Liomba G, Yang LP, Saah AJ, Dallabetta GA, Hoover DR (1995). Infant mortality and maternal vitamin A deficiency during human immunodeficiency virus infection. Clin Infect Dis.

[B7] Semba RD, Miotti PG, Chiphangwi JD, Saah AJ, Canner JK, Dallabetta GA, Hoover DR (1994). Maternal vitamin A deficiency and mother-to-child transmission of HIV-1. Lancet.

[B8] Greenberg BL, Semba RD, Vink PE, Farley JJ, Sivapalasingam M, Steketee RW, Thea DM, Schoenbaum EE (1997). Vitamin A deficiency and maternal-infant transmissions of HIV in two metropolitan areas in the United States. Aids.

[B9] Dreyfuss ML, Fawzi WW (2002). Micronutrients and vertical transmission of HIV-1. Am J Clin Nutr.

[B10] Fawzi WW, Hunter DJ (1998). Vitamins in HIV disease progression and vertical transmission. Epidemiology.

[B11] John GC, Nduati RW, Mbori-Ngacha D, Overbaugh J, Welch M, Richardson BA, Ndinya-Achola J, Bwayo J, Krieger J, Onyango F, Kreiss JK (1997). Genital shedding of human immunodeficiency virus type 1 DNA during pregnancy: association with immunosuppression, abnormal cervical or vaginal discharge, and severe vitamin A deficiency. J Infect Dis.

[B12] Filteau SM, Rollins NC, Coutsoudis A, Sullivan KR, Willumsen JF, Tomkins AM (2001). The effect of antenatal vitamin A and beta-carotene supplementation on gut integrity of infants of HIV-infected South African women. J Pediatr Gastroenterol Nutr.

[B13] Fawzi WW, Msamanga GI, Spiegelman D, Urassa EJ, McGrath N, Mwakagile D, Antelman G, Mbise R, Herrera G, Kapiga S, Willett W, Hunter DJ (1998). Randomised trial of effects of vitamin supplements on pregnancy outcomes and T cell counts in HIV-1-infected women in Tanzania. Lancet.

[B14] Meade MO, Richardson WS (1997). Selecting and appraising studies for a systematic review. Ann Intern Med.

[B15] Altman DG (2001). Systematic reviews of evaluations of prognostic variables. Systematic reviews in Healthcare, Meta-analysis in context.

[B16] Higgins JP, Thompson SG (2002). Quantifying heterogeneity in a meta-analysis. Stat Med.

[B17] Coutsoudis A, Pillay K, Spooner E, Kuhn L, Coovadia HM (1999). Randomized trial testing the effect of vitamin A supplementation on pregnancy outcomes and early mother-to-child HIV-1 transmission in Durban, South Africa. South African Vitamin A Study Group. Aids.

[B18] Kumwenda N, Miotti PG, Taha TE, Broadhead R, Biggar RJ, Jackson JB, Melikian G, Semba RD (2002). Antenatal vitamin A supplementation increases birth weight and decreases anemia among infants born to human immunodeficiency virus-infected women in Malawi. Clin Infect Dis.

[B19] Fawzi WW, Msamanga GI, Hunter D, Renjifo B, Antelman G, Bang H, Manji K, Kapiga S, Mwakagile D, Essex M, Spiegelman D (2002). Randomized trial of vitamin supplements in relation to transmission of HIV-1 through breastfeeding and early child mortality. Aids.

[B20] Friis H, Gomo E, Nyazema N, Ndhlovu P, Krarup H, Kaestel P, Michaelsen KF (2004). Effect of multimicronutrient supplementation on gestational length and birth size: a randomized, placebo-controlled, double-blind effectiveness trial in Zimbabwe. Am J Clin Nutr.

[B21] Joubert G, Steinberg H, van der Ryst E, Chikobvu P (2003). Consent for participation in the Bloemfontein vitamin A trial: how informed and voluntary?. Am J Public Health.

[B22] Fawzi WW, Msamanga G, Hunter D, Urassa E, Renjifo B, Mwakagile D, Hertzmark E, Coley J, Garland M, Kapiga S, Antelman G, Essex M, Spiegelman D (2000). Randomized trial of vitamin supplements in relation to vertical transmission of HIV-1 in Tanzania. J Acquir Immune Defic Syndr.

[B23] Gates S, Brocklehurst P (2004). How should randomised trials including multiple pregnancies be analysed?. Bjog.

[B24] Fawzi W, Msamanga G, Antelman G, Xu C, Hertzmark E, Spiegelman D, Hunter D, Anderson D (2004). Effect of prenatal vitamin supplementation on lower-genital levels of HIV type 1 and interleukin type 1 beta at 36 weeks of gestation. Clin Infect Dis.

[B25] Fawzi WW, Msamanga GI, Spiegelman D, Wei R, Kapiga S, Villamor E, Mwakagile D, Mugusi F, Hertzmark E, Essex M, Hunter DJ (2004). A randomized trial of multivitamin supplements and HIV disease progression and mortality. N Engl J Med.

[B26] Fawzi W (2003). Micronutrients and human immunodeficiency virus type 1 disease progression among adults and children. Clin Infect Dis.

[B27] McClelland RS, Baeten JM, Overbaugh J, Richardson BA, Mandaliya K, Emery S, Lavreys L, Ndinya-Achola JO, Bankson DD, Bwayo JJ, Kreiss JK (2004). Micronutrient Supplementation Increases Genital Tract Shedding of HIV-1 in Women: Results of a Randomized Trial. J Acquir Immune Defic Syndr.

[B28] van Lettow M, Harries AD, Kumwenda JJ, Zijlstra EE, Clark TD, Taha TE, Semba RD (2004). Micronutrient malnutrition and wasting in adults with pulmonary tuberculosis with and without HIV co-infection in Malawi. BMC Infect Dis.

[B29] Kupka R, Msamanga GI, Spiegelman D, Morris S, Mugusi F, Hunter DJ, Fawzi WW (2004). Selenium status is associated with accelerated HIV disease progression among HIV-1-infected pregnant women in Tanzania. J Nutr.

[B30] WMA (2004). Ethical Principles for Medical Research Involving Human Subjects. WORLD MEDICAL ASSOCIATION DECLARATION OF HELSINKI.

[B31] Brocklehurst P, French R (1998). The association between maternal HIV infection and perinatal outcome: a systematic review of the literature and meta-analysis. Br J Obstet Gynaecol.

[B32] Nations U (1998). Universal Declaration of Human Rights. United Nations.

