# Supplementary material for: Vitamin supplementation for prevention of mother-to-child transmission of HIV and pre-term delivery: a systematic review of randomized trial including more than 2800 women
Source: AIDS Res Ther. 2005 May 6;2:4. doi: 10.1186/1742-6405-2-4 (PMC1131887; doi:10.1186/1742-6405-2-4)
Supplement: Additional File 1 — Table 1. Study characteristics. [file 1742-6405-2-4-S1.doc]

### Table 1. Study characteristics

| **Refererence:**  **Name, year, country** | **Intervention** | **Standard care for all participants** | **N of mothers,**  **Gestational period during inclusion** | **N (birth data)** | **Compliance** | **Outcomes measured** | **RR (95% CI)** |
| --- | --- | --- | --- | --- | --- | --- | --- |
| Vitamin A supplementation | | | | | | | |
| Fawzi, 2002, Tanzania  Fawzi, 1998  Tanzania | Preformed Vit A (5,000 IU) + 30mg B-carotene per day, plus 200,000 IU at delivery  Preformed Vit A (5,000 IU) + 30mg B-carotene per day | Women given iron and folate supplements and prophylactic chloroquin.  Infants all given 100, 000 IU of vitamin A at 6 months of age. | 1078,  12-27 weeks  1075,  12-27 weeks | 985  941 | ~89%  Mean 91% | MTCT  Child mortality (12 months)  breastfeeding transmission  Pre-term delivery  Low birth weight  Fetal death | 1.35 (1.11-1.66)  1.08 (0.84-1.39)  1.33 (0.95-1.86)  1.06 (0.83-1.35)  0.89 (0.61-1.29)  0.89 (0.58-1.36) |
| Coutsoudis, 1999, South Africa | Vit A retinyl palmitate (5,000 IU) + B-carotene per day, plus 200,000 IU at delivery | None of the women received ARVs | 728,  1-39 (mean 28) weeks | 661 | NA | MTCT  Preterm delivery  Low birth weight  Child mortality (12 months) | 0.91(0.68-1.24)  0.65(0.44-0.94)  0.86(0.59-1.28)  0.93(0.59-1.48) |
| Kumwenda, 2002, Malawi | Additional Vit A (10,000 IU) per day | Women received 30mg iron and 400ug of folate daily. Women given 30mg retinal equivalent of vit A at 6 weeks pregnancy | 697,  18-28 weeks | 622 | >90% | MTCT  Low birth weight  Child mortality (12 months) | 0.84(0.65-1.08)  0.6(0.39-0.96)  1.08(0.78-1.5) |
| Multivitamin supplementation | | | | | | | |
| Fawzi, 2002, Tanzania  Fawzi, 1998, Tanzania | Multivitamin:  B1 (20mg), B2(25mg), B6(25mg), Niacin (100mg), B12(50ug), Vit. C(500mg), Vit.E(30mg), Folic acid (0.8mg)  Multivitamin: Vitamins: B1 (20mg); B2 (20mg); B6(25mg); Niacin (100mg); B12(50ug); C (500mg); E (30mg); Folic acid (0.8mg) | Women given iron and folate supplements and prophylactic chloroquin.  Infants all given 100, 000 IU of vitamin A at 6 months of age. | 1078,  12-27 weeks  1075,  12-27 weeks | 985  941 | Median ~89%  Median 96% | MTCT  Child mortality (12 months)  Pre-term delivery  Low birth weight  Fetal death | 1.04(0.82-1.32)  0.91(0.17-1.17)  0.86(0.68-1.1)  0.56(0.38-0.82)  0.61(0.39-0.94) |
| Friis, 2004, Zimbabwe | Micronutrient:  Vitamins: A (3000IU); B-carotene (3.5mg); Thiamine (1.5mg); Riboflavin (1.6mg); B-6 (2.2mg); B-12 (4ug); Niacin (17mg); C (80mg); D (10ug); E (10mg). Minerals: Zinc (15mg); Copper (1.2ug); Selenium (65ug) |  | 360 HIV+ mothers (33% of total sample),  22-36 weeks | 352 | One half of the women reported taking >85% | Preterm delivery  Low birth weight | 0.90(0.61-1.35)  1.01(0.62-1.67) |
